# Stage-Aware Swin-Enhanced nnU-Net v2 for Robust Polyp Segmentation in Collaborative Endoscopic Visual Sensing

**DOI:** 10.3390/s26165206

**Published:** 2026-08-17

**Authors:** Yang Bai, Huan Liu

**Affiliations:** School of Computer Science and Technology, Harbin University of Science and Technology, Harbin 150080, China; 2304050102@stu.hrbust.edu.cn

**Keywords:** colon polyp segmentation, nnU-Net v2, Swin Transformer, stage-aware insertion, federated learning, image degradation, robustness, endoscopic sensing

## Abstract

Automatic colon polyp segmentation is important for reliable endoscopic visual sensing. However, segmentation performance can be degraded by ambiguous lesion boundaries, heterogeneous appearance, and image quality variations during acquisition or transmission. This study proposes a stage-aware Swin-enhanced nnU-Net v2 framework, where Swin Transformer blocks are inserted into selected encoder stages while preserving the original nnU-Net v2 pipeline. Different insertion strategies were evaluated on an independent Kvasir-SEG test set, and robustness was further assessed under six synthetic corruption types with three severity levels. The results show that the insertion stage strongly influences the effectiveness of Swin enhancement. Among the evaluated variants, Stage5-Swin achieved the best overall trade-off between segmentation performance, robustness, and computational cost. It achieved the highest Dice scores across all corruption–severity combinations while maintaining comparable external-domain performance on CVC-ClinicDB. Additional FedAvg experiments demonstrated the compatibility of the proposed architecture with collaborative training workflows. Resource analysis further quantified the computational and communication overhead. The findings indicate that middle-to-deep encoder insertion provides a favorable balance between contextual modeling, spatial representation, and efficiency for robust endoscopic segmentation. However, improvements were metric- and corruption-dependent, and moderate overexposure revealed a Precision–Recall trade-off; practical deployment also remains to be validated.

## 1. Introduction

Colonoscopy is a primary tool for colorectal cancer screening and early diagnosis. Accurate identification and boundary delineation of polyps directly affect subsequent resection decisions, pathological evaluations, and clinical risk assessments. With advances in endoscopic equipment, edge-computing platforms, and computer-aided diagnosis systems, automatic segmentation methods for endoscopic images have become an important research direction in intelligent medical sensing. However, in real clinical settings, polyps often exhibit heterogeneous appearances, with substantial inter-case variations in size, shape, color, texture, and contrast with the surrounding mucosa. Motion blur, specular highlights, low illumination, overexposure, and compression artifacts during acquisition or transmission can further degrade image quality and increase the generalization challenge for automatic segmentation models.

In medical image segmentation, U-Net has long served as a foundational architecture [1], while nnU-Net and its subsequent validation studies emphasize the importance of strong self-configuring baselines, standardized preprocessing, training strategies, and rigorous evaluation protocols [2,3]. Public colonoscopy polyp datasets, including Kvasir-SEG, as used in this study, and CVC-ClinicDB, as introduced in prior validation work, have supported reproducible benchmarking of endoscopic segmentation algorithms [4,5]. Recent polyp segmentation networks, including PraNet, HarDNet-MSEG, UACANet, and Polyp-PVT, have improved boundary modeling, context aggregation, uncertainty-aware attention, or Transformer-based representation learning for challenging colonoscopic images [6,7,8,9]. However, these methods mainly focus on designing specialized segmentation architectures, whereas this study investigates the influence of Transformer insertion position within a strong and standardized nnU-Net v2 Baseline. These studies suggest that accurate polyp segmentation requires both local texture extraction and global context modeling of lesions and ambiguous boundaries.

Recent studies also indicate that polyp segmentation research is moving from single-test-set accuracy optimization to more practical evaluation dimensions, including cross-dataset generalization, real-time applicability, and computational efficiency. Transformer-based models such as Polyp-PVT and ColonFormer have demonstrated the value of hierarchical attention and multi-level representation learning for colonoscopic lesion segmentation [9,10]. Recent CNN–Transformer hybrid designs further indicate that global semantic modeling should be balanced with local boundary preservation and computational cost [11]. At the same time, community benchmarks and challenge-based analyses suggest that high segmentation accuracy alone is insufficient for clinical translation; validation should also consider generalizability, processing speed, and deployment robustness in dynamic colonoscopy conditions [12,13]. These observations motivate the present work to evaluate not only clean test accuracy but also degradation robustness and resource-aware efficiency.

Convolutional networks primarily rely on local receptive fields and layer-wise downsampling to capture semantic context. When polyps have textures similar to the surrounding mucosa, blurred boundaries, or severe highlight occlusion, relying only on local convolutional features may lead to omissions, boundary expansion, or false positives. Swin Transformer captures medium- to long-range dependencies through window-based self-attention and shifted windows while maintaining computational efficiency [14]. Transformer-based medical segmentation methods, including TransUNet, UNETR, Swin-Unet, Swin UNETR, TransFuse, and MedT, further demonstrate the value of combining attention-based context modeling with U-shaped or hybrid segmentation architectures [15,16,17,18,19,20]. Unlike approaches that replace the entire encoder, the proposed design inserts a Swin module after a selected stage of the existing nnU-Net v2 encoder. This strategy supplements global contextual modeling while retaining the self-configuring advantages of nnU-Net v2. Robustness evaluation under common image corruptions and quality degradation is also important for assessing whether segmentation models can remain reliable under acquisition and transmission artifacts [21,22].

This study further considers privacy protection and cross-institutional collaboration in intelligent endoscopic perception. Endoscopic images are highly privacy-sensitive, and it is often difficult for medical institutions to directly share raw data. Federated learning provides a viable path for cross-institutional model development through a local training and server aggregation workflow [23,24,25]. In medical image analysis, privacy-preserving federated segmentation and recent survey studies further indicate that collaborative training without direct cross-site data sharing is an important direction for privacy-aware clinical AI development [26,27]. However, federated medical segmentation remains affected by feature shift, domain heterogeneity, and generalization gaps across institutions [28,29]. Therefore, the federated experiment is positioned as a supplementary feasibility analysis. Its purpose is to determine whether the proposed architecture can be integrated into a FedAvg workflow, rather than to provide primary evidence of segmentation superiority.

In intelligent communication, computing, and sensing scenarios, an endoscopic AI system can be regarded as a visual sensing agent. It perceives continuously changing endoscopic scenes and performs local or edge-side inference. It may also collaborate with other clinical nodes through model training without directly exchanging raw images. In this setting, three requirements become important: robust sensing under degraded visual inputs, collaborative learning without direct raw-image exchange, and efficient computation under edge-device constraints. Therefore, this study does not address multimodal sensing fusion; instead, it focuses on a narrower but practical scenario of robust, collaborative, and resource-aware visual sensing for colonoscopic polyp segmentation. This scope aligns with the Special Issue topics of federated learning, collaborative model training, edge AI, distributed computing frameworks, and sensing technologies.

The main contributions are summarized as follows.

(1) This study systematically investigates the stage sensitivity of Swin Transformer insertion in nnU-Net v2. The analysis focuses on clean test behavior, degradation robustness, and computational cost rather than broad accuracy superiority over the Baseline.
(2) Eighteen degraded test sets were constructed from the same independent Kvasir-SEG test set by applying six corruption types at mild, moderate, and severe levels. This severity-aware protocol enables both corruption-specific evaluation and corruption–severity curve analysis, allowing us to examine whether the relative conclusions remain stable as image quality progressively deteriorates.
(3) An additional external evaluation was conducted on CVC-ClinicDB to examine cross-dataset generalization from Kvasir-SEG to an unseen colonoscopy dataset. This experiment assesses whether Stage5-Swin remains stable under external-domain transfer, rather than claiming universal superiority over the Baseline.
(4) A supplementary four-client IID FedAvg experiment examines whether the Swin-embedded architecture can be incorporated into a collaborative training workflow. Parameters, FLOPs, FPS, GPU memory, model size, and communication overhead are also analyzed to provide a resource-aware assessment for potential edge-sensing scenarios.

## 2. Materials and Methods

### 2.1. Dataset and Splitting Strategy

The experimental data were obtained from the Kvasir-SEG dataset, which contains colonoscopy polyp images and corresponding pixel-level annotations and is widely used in polyp detection and segmentation research [4]. To align with the training workflow of nnU-Net v2, the data were organized into the standard nnU-Net v2 format, and a fixed splitting scheme was adopted for training, validation, and testing. Specifically, a total of 900 images were used for training and validation, with an additional 100 images set aside as an independent clean test set. Under the fold 0 configuration, the training set consisted of 720 images and the validation set of 180 images. The independent test set was not used for training or validation but was reserved for a unified comparison across different models to ensure consistency and comparability in result evaluation. 

The 2D configuration of nnU-Net v2 was used with a patch size of 640 × 640 and a batch size of 8. All other training strategies followed the default nnU-Net v2 settings. To ensure a fair comparison among different models, the Baseline and all Swin variants used the same plans, split files, and random seed, with the seed set to 42. All models were trained and evaluated under the same hardware and software environment. Evaluation metrics included Dice, IoU, Precision, Recall, and HD95. The dataset organization, splitting strategy, nnU-Net v2 configuration, randomness control, evaluation metrics, and implementation environment are summarized in Table 1.

### 2.2. Baseline nnU-Net v2 and Stage-Aware Swin Insertion

The Baseline model uses the 2D PlainConvUNet architecture automatically configured by nnU-Net v2. To preserve the original nnU-Net v2 training and inference pipeline, this study does not replace the encoder, decoder, skip connections, or deep supervision mechanism. Instead, a Swin Transformer block is appended after a selected convolutional encoder stage as a feature refinement module. In this way, the proposed variants maintain the strong self-configuring ability of nnU-Net v2 while introducing attention-based contextual modeling at specific encoder stages.

Let the output feature map of the selected encoder stage be $X_{s}\in {\mathbb{R}}^{B\times C_{s}\times H_{s}\times W_{s}}$, where *B* denotes the batch size, $C_{s}$ denotes the number of channels, and $H_{s}$ and $W_{s}$ denote the spatial height and width of the feature map at stage s. For the Swin-enhanced variants in this study, the channel dimension of the inserted stage is $C_{s}=512$. Under the 640×640 input patch configuration, the three tested insertion stages correspond to feature map sizes of approximately 40×40, 20×20, and 5×5.

Before self-attention is applied, the feature map $X_{s}$ is converted into a token sequence. Specifically, the spatial dimensions $H_{s}$ and $W_{s}$ are flattened into $N_{s}=H_{s}W_{s}$ tokens, and the tensor layout is transformed from $B\times C_{s}\times H_{s}\times W_{s}$ to $T_{s}\in {\mathbb{R}}^{B\times N_{s}\times C_{s}}$. This token representation is required by the window-based self-attention operation in the Swin Transformer block. After the attention-based refinement, the token sequence is reshaped back to the original feature map format, producing $Y_{s}\in {\mathbb{R}}^{B\times C_{s}\times H_{s}\times W_{s}}$, which is then passed to the subsequent layers of the original nnU-Net v2 encoder–decoder network.

The inserted Swin block consists of LayerNorm, window-based multi-head self-attention, shifted window information exchange, a multi-layer perceptron (MLP), and residual connections. Its main operations can be summarized as follows:

(1) $$T_{s}^{\prime }=T_{s}+\mathrm{W-MSA}(\mathrm{LN}(T_{s}))$$

(2) $$T_{s}^{\prime \prime }=T_{s}^{\prime }+\mathrm{MLP}(\mathrm{LN}(T_{s}^{\prime }))$$

where LN denotes LayerNorm, W-MSA denotes window-based multi-head self-attention, and MLP denotes the feed-forward multilayer perceptron. The first residual connection adds the attention output back to the input token sequence, while the second residual connection adds the MLP output back to the attention-refined sequence. In alternating Swin layers, the shifted window mechanism enables information exchange across neighboring windows, thereby expanding contextual interaction beyond a single local window while avoiding the high computational cost of full global self-attention [14].

**Figure 1:**
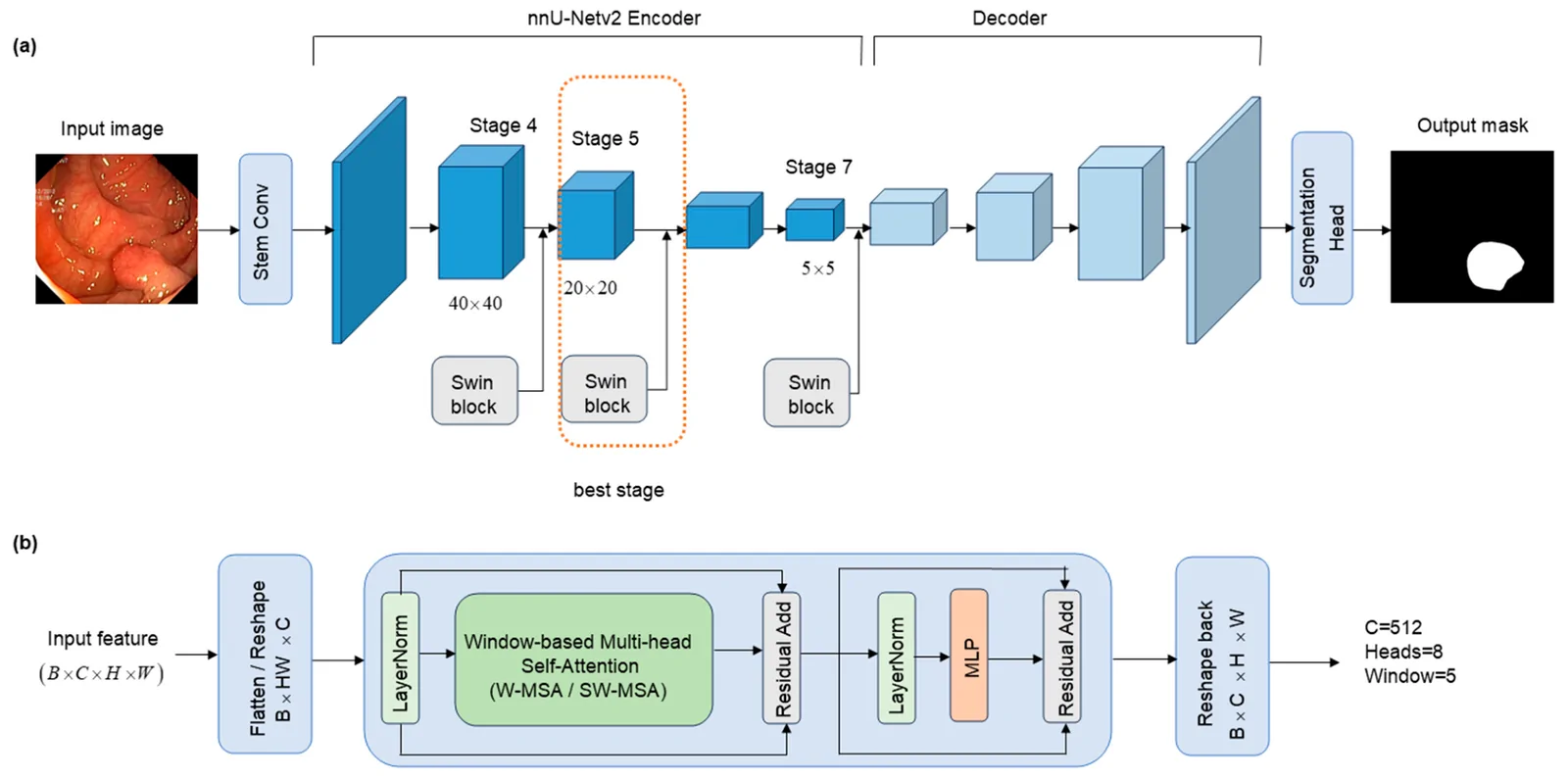
Architecture of the proposed stage-aware Swin-enhanced nnU-Net v2: (**a**) candidate Swin insertion stages in the nnU-Net v2 encoder, including Stage 4, Stage 5, and Stage 7; (**b**) the inserted Swin block, including flatten/reshape, LayerNorm, window-based or shifted window multi-head self-attention, MLP, and residual connections. Example endoscopic images are from the publicly available Kvasir-SEG dataset [4].

As shown in Figure 1 and summarized in Table 2, this study compares three Swin insertion positions: Stage4-Swin, Stage5-Swin, and Stage7-Swin. The candidate insertion stages were selected based on a balance between spatial resolution, semantic abstraction, and computational feasibility. Earlier encoder stages (Stages 0–3) were not included because their feature maps maintain substantially higher spatial resolutions, resulting in a much larger number of tokens and significantly increased self-attention computation. Stage6 and Stage7 represent deeper semantic representations close to the bottleneck. Since Stage6 and Stage7 share similar bottleneck-level characteristics, Stage7 was selected as the representative extreme case to evaluate whether excessive spatial compression limits the effectiveness of Swin-based attention. Therefore, Stage4, Stage5, and Stage7 were selected as representative relatively high-resolution, intermediate, and bottleneck-level insertion locations.

Stage4-Swin is inserted at a relatively high-resolution encoder stage, where the feature map contains more spatial tokens. This setting may help preserve fine boundary details, but it also increases the attention-related computational burden and may affect mid-level convolutional feature extraction. Stage7-Swin is inserted near the bottleneck, where the feature map is highly compressed and contains only a small number of spatial tokens. Although this design is computationally cheaper, the limited spatial resolution may reduce the benefit of window-based self-attention for spatial feature refinement. Stage5-Swin represents an intermediate design that operates on a middle-to-deep feature representation with sufficient semantic abstraction while still retaining a meaningful spatial token grid. Therefore, it is expected to provide a more balanced compromise among contextual modeling, spatial resolution, and computational efficiency.

The purpose of this stage-aware design is not to claim that Transformer modules are inherently superior to convolutional features. Instead, the aim is to examine whether the insertion stage of a Swin Transformer block affects the segmentation accuracy, degradation robustness, and computational efficiency of a strong nnU-Net v2 Baseline. This design also allows the original nnU-Net v2 framework to remain largely unchanged, making the comparison among different insertion stages more controlled and interpretable. Therefore, the conclusions regarding stage selection are limited to the evaluated insertion locations, and further investigation of other encoder stages remains a potential direction for future work.

### 2.3. External CVC-ClinicDB Evaluation Protocol

To further evaluate cross-dataset generalization, an external evaluation was conducted on CVC-ClinicDB using all 612 images as the external test set. The Baseline and Stage5-Swin models trained on Kvasir-SEG were directly applied to CVC-ClinicDB without additional fine-tuning, retraining, or parameter adjustment. The dataset was converted into the same nnU-Net-compatible inference format used in the Kvasir-SEG evaluation, with test images placed in imagesTs and the corresponding binary masks placed in labelsTs. The same preprocessing, prediction, postprocessing, and evaluation scripts were used for both models to ensure a fair comparison. The evaluation metrics included Dice, IoU, Precision, Recall, and HD95. This external experiment was designed to examine whether the proposed Stage5-Swin variant remains stable under cross-dataset transfer, rather than to establish a comprehensive external dataset superiority claim.

### 2.4. Robustness Test Sets with Multiple Corruption Severities

To evaluate the reliability of endoscopic visual sensing under progressively degraded acquisition and transmission conditions, severity-controlled corrupted test sets were generated from the same 100-image independent Kvasir-SEG test set. The segmentation annotations were kept unchanged, and only the input images were modified. Six corruption types were considered: Gaussian noise, blur, low illumination, overexposure, JPEG compression, and low-resolution resampling. Each corruption was applied at three severity levels—mild, moderate, and severe—resulting in 18 degraded test sets. The same degraded images were used to evaluate the Baseline, Stage4-Swin, and Stage5-Swin models, thereby enabling paired and directly comparable evaluation across models.

The corruption intensities were selected to represent progressively increasing but visually interpretable degradation. For Gaussian noise, the standard deviation was set to σ=5, 10, and 20 on the 0–255 pixel value scale. Blur was generated using box blur radii of 1, 2, and 4 pixels, corresponding to effective kernel sizes of approximately 3×3, 5×5, and 9×9. Low illumination was simulated using luminance scaling factors of 0.8, 0.6, and 0.4, while overexposure was simulated using factors of 1.2, 1.4, and 1.8. JPEG compression quality was set to 50, 30, and 10, where a lower quality value represents stronger compression. For low-resolution degradation, the images were first downsampled by factors of 0.75, 0.50, and 0.25 and subsequently resized back to their original dimensions using bilinear interpolation.

The moderate levels matched the settings used in the original robustness experiment. Mild and severe levels were added to determine whether the model rankings remained stable under weaker and stronger perturbations. All corrupted datasets were generated using a fixed random seed of 42. In particular, the same noise realization for each test image and severity level was evaluated by all models, preventing model-specific differences in the generated perturbations. The selected intensity ranges were intended to span visible quality deterioration without making all images diagnostically unrecognizable. Because these corruptions remain synthetic approximations of acquisition and transmission artifacts, they are interpreted as controlled stress tests rather than complete simulations of clinical image quality variation.

### 2.5. Federated Learning Protocol

To examine whether the proposed architecture can be integrated into a federated collaborative model training workflow, a supplementary FedAvg experiment was conducted. The 900 Kvasir-SEG training images were divided into four local subsets under an independent and identically distributed (IID) setting. Each subset represented one virtual clinical node and contained 225 images. The federated experiment employed the FedAvg framework [23]. At the beginning of each communication round, the server distributed the current global model to all clients. Each client then trained the model independently on its local data. After local training, only the updated model weights were uploaded to the server. The server computed a sample-size-weighted average to obtain the global model for the next round.

The global aggregation formula for FedAvg is:

(3) $$w_{t+1}=\sum_{k=1}^{K}\frac{n_{k}}{n}w_{t+1}^{\left(k\right)},$$

where *K* is the number of clients, $n_{k}$ is the number of training samples for the *k*th client, n is the total number of training samples, and $w_{t+1}^{\left(k\right)}$ denotes the model parameters after local training by the kth client. The formal experiment used 4 clients, 20 rounds, local_epochs = 1, iters_per_epoch = 50, and val_iters = 10. During aggregation, only the network weights were averaged; the optimizer, logger, and current_epoch were not restored to prevent cross-contamination of training states among different clients. To ensure the stability of the Swin module during the federated training cycle, the Swin-FedAvg experiment used standard PyTorch eager mode and disabled dynamic compilation via torch.compile. It should be noted that the federated section of this paper is primarily used to verify engineering compatibility and training trends; core performance conclusions are still based on centralized independent tests and degradation robustness experiments. In the supplementary FedAvg experiments, the same protocol was applied to the FedAvg Baseline, FedAvg Stage4-Swin, and FedAvg Stage5-Swin models to compare their validation performance trajectories under an identical collaborative training setting.

### 2.6. Evaluation Metrics and Statistical Analysis

All centralized models and the federated global model used the same prediction pipeline and a unified scoring script. Region-overlap metrics included Dice and IoU. Precision and Recall were used to characterize false positive and false negative tendencies. HD95 was used to quantify boundary error, with lower values indicating better boundary agreement. The metrics are calculated as follows:

(4) $$\begin{array}{cc} \mathrm{Dice}=\frac{2\mathrm{TP}}{2\mathrm{TP}+\mathrm{FP}+\mathrm{FN}}, & \mathrm{IoU}=\frac{\mathrm{TP}}{\mathrm{TP}+\mathrm{FP}+\mathrm{FN}},\\ \mathrm{Precision}=\frac{\mathrm{TP}}{\mathrm{TP}+\mathrm{FP}}, & \mathrm{Recall}=\frac{\mathrm{TP}}{\mathrm{TP}+\mathrm{FN}}. \end{array}$$

In the robustness analysis, performance variation metrics were further introduced to characterize the extent of the impact of degraded conditions relative to clean conditions. Specifically, Dice Drop and IoU Drop were used to measure the decline in overlap performance, while HD95 Increase was used to describe the magnitude of the increase in boundary error.

(5) $$\begin{array}{cc} \mathrm{Dice}\;\mathrm{Drop}\;\;\;= & \;\;\;{\mathrm{Dice}}_{\mathrm{clean}}-{\mathrm{Dice}}_{\mathrm{degraded}},\\ \mathrm{IoU}\;\mathrm{Drop}\;\;\;\;= & \;\;\;\;{\mathrm{IoU}}_{\mathrm{clean}}-{\mathrm{IoU}}_{\mathrm{degraded}},\\ \mathrm{HD}95\;\mathrm{Increase}\;= & \;{\mathrm{HD}95}_{\mathrm{degraded}}-{\mathrm{HD}95}_{\mathrm{clean}}. \end{array}$$

To compare the Baseline and Stage5-Swin at the per-image level, two-sided paired Wilcoxon signed-rank tests were performed [30]. Predictions from the two models were paired by case_id so that each comparison involved the same test image under the same evaluation condition. Statistical significance was defined as a raw *p*-value < 0.05. The analysis included the clean test set and the six moderate-severity degradation settings, with Dice, IoU, Precision, Recall, and HD95 evaluated separately for each setting. No multiple comparison correction was applied because the 35 comparisons were treated as exploratory analyses; therefore, the raw *p*-values are reported and interpreted cautiously rather than as confirmatory evidence. The additional mild- and severe-level degradation experiments were used to characterize severity sensitivity and were analyzed descriptively rather than inferentially. Complete results for all 35 comparisons are provided in Supplementary Table S1, while the statistically significant moderate-severity results are presented in a later section.

## 3. Results

### 3.1. Centralized Stage-Aware Ablation on the Independent Clean Test Set

Table 3 presents the centralized evaluation results of the Baseline, Stage4-Swin, Stage5-Swin, and Stage7-Swin on a 100-image independent clean test set. Overall, Stage5-Swin achieved the highest mean Dice, IoU, Precision, and Recall among the tested insertion-stage variants, and it also showed the lowest mean HD95. However, the absolute gains over the nnU-Net v2 Baseline were small. Dice increased from 0.8838 to 0.8876, and IoU increased from 0.8222 to 0.8245. Therefore, these clean test results should not be interpreted as strong evidence of accuracy superiority but rather as evidence that the insertion stage affects the behavior of Swin-enhanced nnU-Net v2. In contrast, inserting it too early (Stage4) or too deep (Stage7) fails to yield consistent benefits.

Compared with the Baseline, Stage5-Swin improved Dice by 0.0038 and IoU by 0.0023, while HD95 decreased from 43.1908 to 41.7450. Stage4-Swin achieved slightly higher Recall than the Baseline but showed no advantage in Dice, IoU, or HD95. Stage7-Swin produced the weakest overall results. The limited number of tokens on its 5 × 5 bottleneck feature map may restrict the spatial modeling benefits of Swin self-attention.

**Figure 2:**
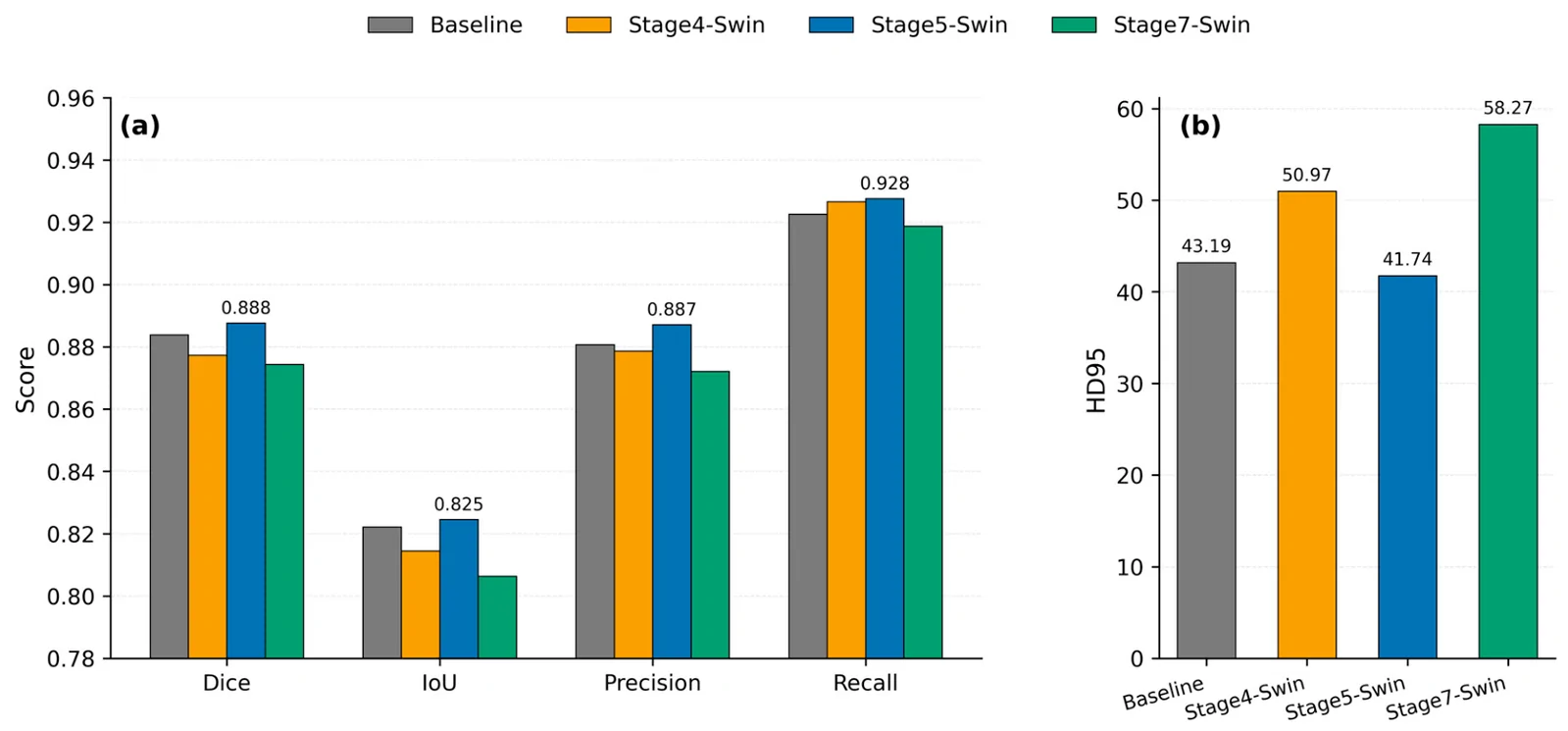
Centralized performance comparison of models with different insertion stages on 100 independent clean test images. (**a**) Dice, IoU, Precision, and Recall; (**b**) HD95.

To visualize the effect of different Swin insertion stages on the performance of the clean test set, Figure 2 shows both the area overlap metrics and the boundary distance metrics. Figure 2a compares the differences in Dice, IoU, Precision, and Recall between the Baseline, Stage4-Swin, Stage5-Swin, and Stage7-Swin models, while Figure 2b shows HD95 separately, where lower values indicate better boundary consistency. This figure further illustrates that Stage5-Swin demonstrates the most balanced performance among the tested insertion-stage variants.

### 3.2. Robustness Across Corruption Types and Severity Levels

Table 4 first summarizes the average performance across the six moderate-severity degraded test sets. These moderate settings correspond to the corruption intensities used in the original robustness experiment. Stage5-Swin achieved the highest average Dice and IoU under these conditions, with values of 0.8843 and 0.8201, respectively.

In absolute terms, Stage5-Swin achieved the best degraded-average Dice, IoU, and Precision. Stage4-Swin had the smallest HD95 Increase, but its clean HD95 and degraded average HD95 were both substantially higher than those of Stage5-Swin. This observation indicates that focusing solely on the relative change from the clean condition may mask differences in absolute boundary error. Therefore, after considering region overlap, boundary error, and relative decline, Stage5-Swin is the more balanced solution. 

Table 5 reports the Dice scores of the three models across all 18 corruption–severity combinations. Stage5-Swin achieved the highest Dice in every combination. This ranking remained unchanged across the mild, moderate, and severe levels of all six corruption types. Therefore, the observed Dice advantage was not restricted to a single manually selected intensity.

As expected, stronger corruption generally resulted in lower segmentation accuracy, although small non-monotonic variations were observed in a few mild-to-moderate settings. For Stage5-Swin, Dice decreased from 0.888 to 0.874 for Gaussian noise, from 0.888 to 0.872 for blur, and from 0.885 to 0.854 for JPEG compression when severity increased from mild to severe. The largest degradation occurred under overexposure, where Dice decreased from 0.881 at the mild level to 0.840 at the severe level. In contrast, low-illumination and low-resolution degradation produced relatively smaller Dice variations across the selected severity ranges.

These results indicate that all models are affected by sufficiently strong corruption, particularly severe overexposure and JPEG compression. Nevertheless, the relative ordering of the three models remains stable across severity levels, with Stage5-Swin consistently outperforming both the Baseline and Stage4-Swin. Therefore, the robustness conclusion is not dependent on the original single moderate-corruption setting.

**Figure 3:**
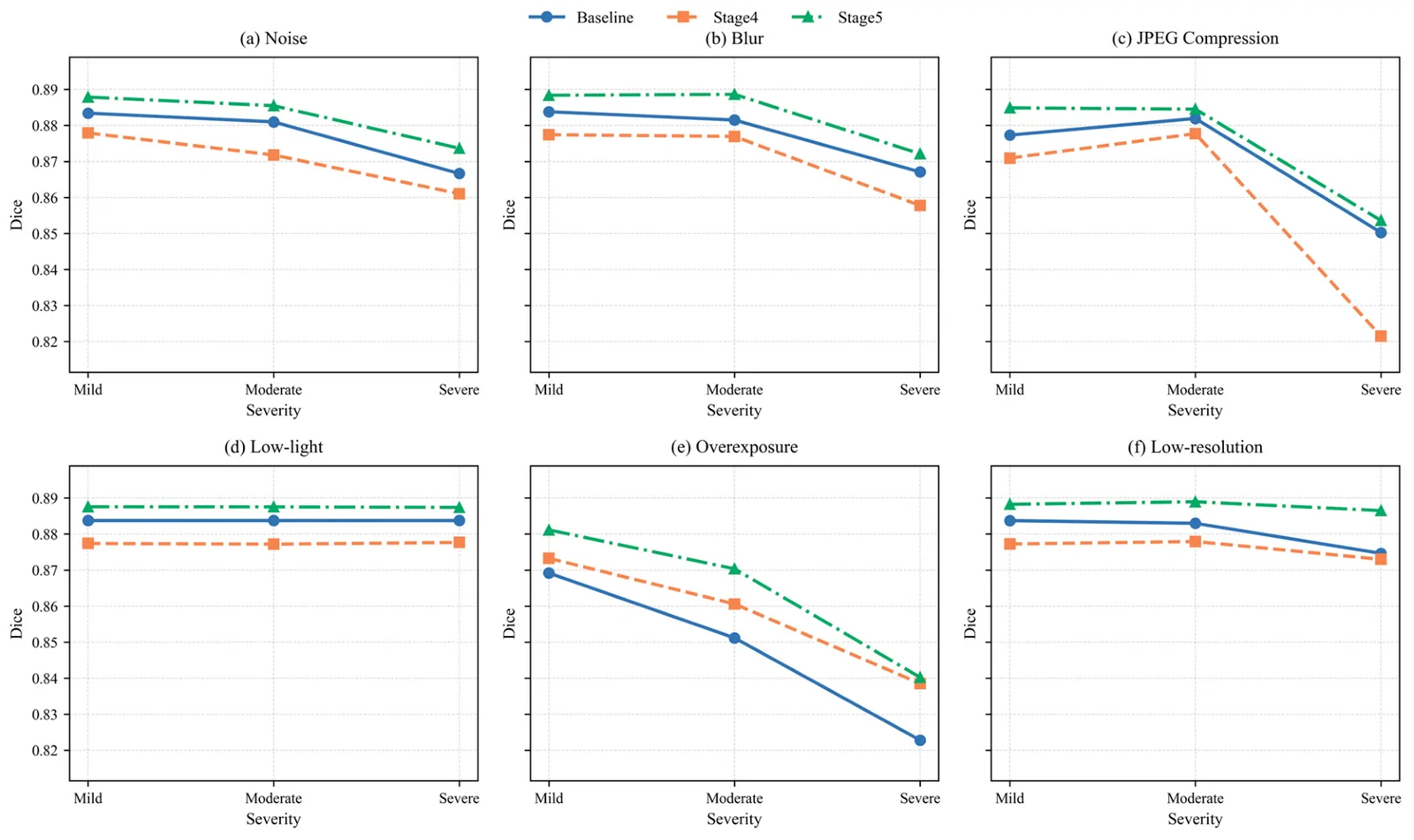
Corruption–severity curves of segmentation performance under six image degradation types. Each panel shows Dice variation from mild to severe corruption for the Baseline, Stage4-Swin, and Stage5-Swin models.

To further visualize the relationship between corruption strength and segmentation performance, Figure 3 presents corruption–severity curves for the six degradation types. Each curve shows the Dice variation from mild to severe corruption for the Baseline, Stage4-Swin, and Stage5-Swin models. The results demonstrate that although Dice generally decreases under stronger degradation, the relative ranking among models remains unchanged, with Stage5-Swin achieving the highest performance across all severity levels.

These degradation results indicate that Stage5-Swin maintains a stable Dice-based relative advantage across the evaluated synthetic corruption scenarios. However, the robustness findings were not uniform across all metrics and conditions. Improvements in Dice or IoU do not necessarily imply that HD95 will improve for every corruption setting or every individual image because HD95 is particularly sensitive to localized contour deviations and spatially distant boundary outliers. Therefore, region-overlap performance, aggregate boundary error, and per-image boundary behavior should be interpreted separately. Overall, the results suggest that middle-to-deep contextual modeling can help preserve lesion-level segmentation under controlled image degradation, while localized boundary errors may still occur in individual cases.

**Figure 4:**
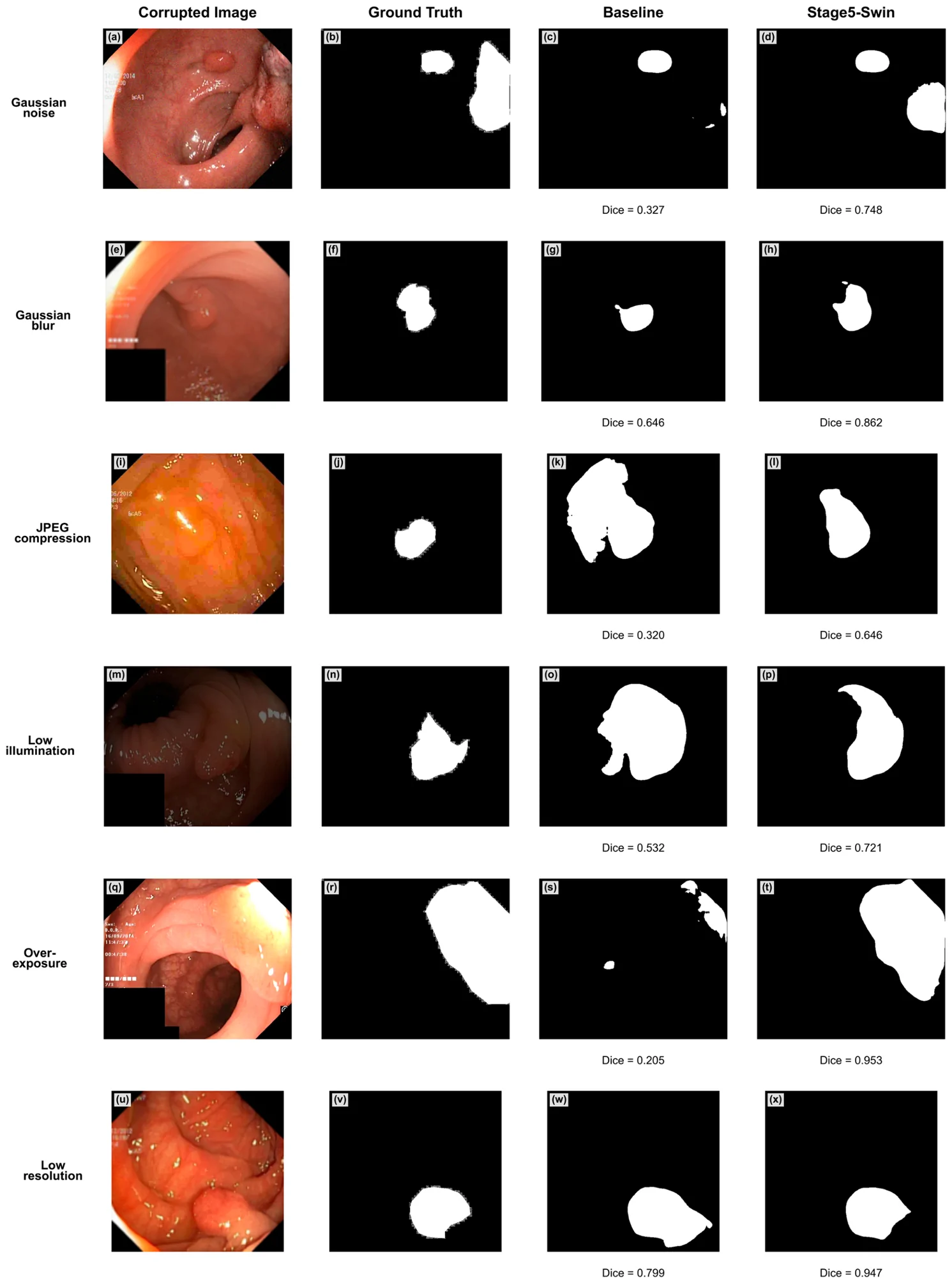
Representative segmentation results under severe image corruptions. Rows correspond to severe Gaussian noise (**a**–**d**), Gaussian blur (**e**–**h**), JPEG compression (**i**–**l**), low illumination (**m**–**p**), overexposure (**q**–**t**), and low-resolution degradation (**u**–**x**). From left to right, each row shows the corrupted input image, ground truth mask, Baseline prediction, and Stage5-Swin prediction. Dice scores are shown below the corresponding predictions.

To complement the aggregate severity analysis, Figure 4 presents one representative severe-degradation case for each corruption type. The cases were selected from the independent test set to illustrate visually discernible differences between the Baseline and Stage5-Swin while avoiding repeated use of the same image across corruption types. These examples qualitatively show that Stage5-Swin can better preserve lesion structures under several severe-degradation conditions, such as blur, JPEG compression, and low-resolution distortion. However, these cases are only visual illustrations and do not represent independent statistical evidence. The overall robustness conclusions are supported by the complete severity evaluation in Table 5 and Figure 3.

The qualitative results are consistent with the full-test-set severity curves but also reveal the remaining limitations. In particular, severe overexposure can remove or saturate local appearance cues, while random noise may still introduce boundary perturbations. Therefore, the representative cases provide visual examples of success and failure patterns rather than independent statistical evidence.

### 3.3. Paired Statistical Analysis

Table 6 summarizes the statistically significant differences between the Baseline and Stage5-Swin under the six moderate-severity degradation conditions, based on two-sided paired Wilcoxon signed-rank tests. Complete results for the clean condition and all six moderate-severity conditions are reported in Supplementary Table S1. None of the five clean-condition comparisons reached the raw significance threshold of *p* < 0.05, although the Stage5-Swin means were numerically better for all five metrics. Under moderate blur, Stage5-Swin showed significantly higher Dice, IoU, and Precision and significantly lower HD95 than the Baseline. Significant differences were also observed for Precision under low-resolution degradation, HD95 under Gaussian noise, and both Precision and Recall under overexposure. Because no multiple comparison correction was applied, these raw *p*-values are interpreted as exploratory rather than confirmatory evidence.

The statistical findings were metric- and corruption-dependent rather than uniformly favorable across all comparisons. Under moderate blur, Stage5-Swin showed the clearest pattern of improvement, with significant differences in Dice, IoU, Precision, and HD95. Under Gaussian noise, Stage5-Swin reduced mean HD95 from 44.2674 to 41.0420 (raw *p* = 0.0268), indicating improved aggregate boundary accuracy under the moderate noise setting, whereas the differences in Dice, IoU, Precision, and Recall were not statistically significant. Under low-resolution degradation, only Precision reached the raw significance threshold. Under moderate overexposure, Precision increased from 0.8478 to 0.8792 (raw *p* = 0.0316), whereas Recall decreased from 0.9189 to 0.9028 (raw *p* = 0.0404). This opposite behavior indicates a Precision–Recall trade-off: Stage5-Swin produced fewer false positive predictions but omitted more lesion pixels under moderate overexposure. The remaining comparisons did not reach the raw *p* < 0.05 threshold, and all results should be interpreted cautiously because no multiple comparison correction was applied.

**Figure 5:**
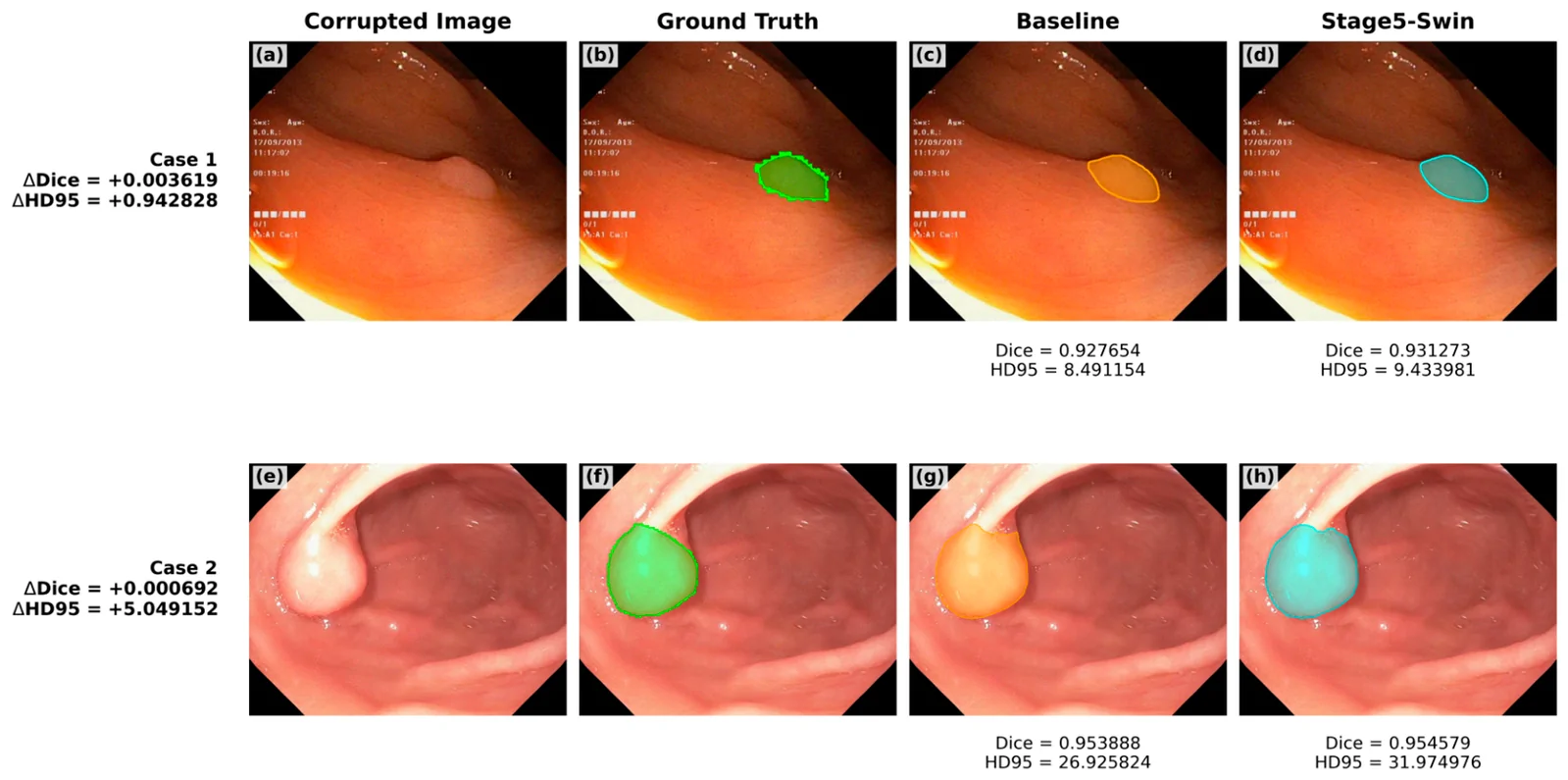
Representative Gaussian noise cases illustrating the relationship between region overlap and boundary distance. Panels (**a**–**d**) show Case 1 and panels (**e**–**h**) show Case 2. From left to right, each row presents the corrupted input image, ground truth segmentation, Baseline prediction, and Stage5-Swin prediction. Dice and HD95 values are reported below the corresponding predictions.

Although Stage5-Swin achieved a significantly lower mean HD95 under moderate Gaussian noise, this aggregate improvement was not uniform across all individual cases. Per-image inspection identified a small number of cases in which Stage5-Swin achieved slightly higher Dice but also higher HD95 than the Baseline. Figure 5 presents two such representative cases from the current moderate-Gaussian noise evaluation. The cases were selected from images satisfying the predefined condition that Stage5-Swin had both a higher Dice and a higher HD95 than the Baseline; one case with a mild HD95 Increase and one with a larger increase were used to illustrate different degrees of overlap–boundary discrepancy. In Case 1, the improvement in overall foreground overlap is accompanied by a mild increase in boundary distance error. In Case 2, the principal lesion region remains accurately segmented, but a localized contour deviation or spatially distant boundary error produces a substantially larger HD95. These examples show that Dice and HD95 capture complementary aspects of segmentation quality: Dice mainly reflects global foreground overlap, whereas HD95 is more sensitive to localized or distant boundary outliers. Therefore, a prediction can achieve slightly better overall overlap while still exhibiting a worse HD95 in an individual case.

Therefore, Stage5-Swin should not be interpreted as uniformly superior across all metrics, degradation conditions, or individual cases. Its main advantage is reflected in stable Dice-based performance across corruption severities and in aggregate HD95 improvements under moderate blur and Gaussian noise. Nevertheless, Figure 5 shows that localized boundary outliers may still occur in individual noise-degraded images despite favorable average results. In addition, the reduced Recall under moderate overexposure indicates a condition-specific risk of missed lesion pixels. These findings support a joint interpretation of region overlap, boundary distance, and lesion coverage rather than a uniform claim of boundary robustness.

### 3.4. FedAvg Evaluation of Collaborative Training

In the federated learning section, FedAvg Baseline, FedAvg Stage4-Swin, and FedAvg Stage5-Swin were trained using a four-client, 20-round IID FedAvg protocol under the same collaborative protocol. According to federated_training_metrics.csv, the highest mean validation pseudo-Dice values were obtained at round 18 for the Baseline, round 16 for Stage4-Swin, and round 19 for Stage5-Swin. Specifically, the best validation pseudo-Dice values were 0.6831 for the Baseline, 0.6543 for Stage4-Swin, and 0.6774 for Stage5-Swin. The final communication round was round 19 for all models. The complete FedAvg protocol, including the number of clients, communication rounds, local training schedule, and selected best/final rounds, is summarized in Table 7.

Based on these selected checkpoints, Table 8 reports the clean test performance of the FedAvg global models for both the best and final rounds.

Based on the clean test results, FedAvg Baseline outperforms FedAvg Stage4-Swin in Dice, IoU, Precision, and Recall, especially in the best-checkpoint comparison. In the final-round comparison, Stage4-Swin obtains a lower HD95 than the Baseline (110.4066 vs. 118.7643), but this isolated boundary distance observation should not be interpreted as evidence of an overall federated performance advantage. Therefore, the FedAvg experiment is used here to demonstrate compatibility with federated learning and collaborative model training, rather than to claim a federated performance advantage of the Swin variant. The main performance conclusions remain based on centralized independent testing and degradation robustness evaluation, while the FedAvg results provide supplementary evidence that the proposed architecture can participate in a multi-client training and aggregation workflow.

**Figure 6:**
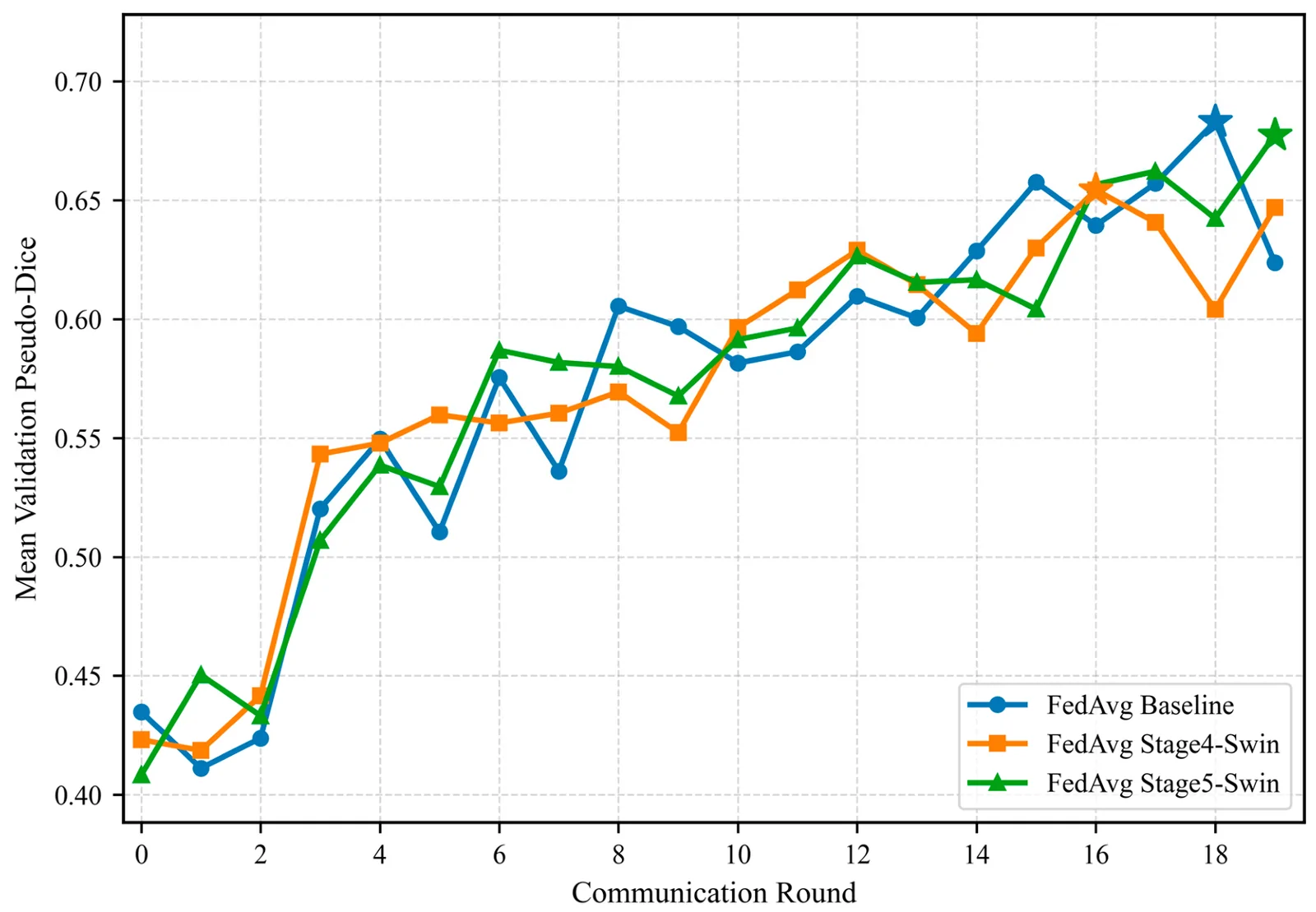
Validation performance trends of FedAvg Baseline, Stage4-Swin, and Stage5-Swin over 20 IID communication rounds. Asterisks indicate the best validation pseudo-Dice checkpoint for each model.

To complement the quantitative test results, Figure 6 shows the validation set performance trends of the Baseline, Stage4-Swin, and Stage5-Swin during the 20-round IID FedAvg training process. All three models show increasing validation pseudo-Dice trends during the early communication rounds, although performance fluctuations remain near the end of the training process. Therefore, the 20-round FedAvg experiment is interpreted as a short-horizon optimization trend analysis rather than evidence of full convergence. However, the federated and centralized settings optimize under different training dynamics; therefore, the pseudo-Dice values are used only for convergence analysis within the federated protocol and should not be directly compared with centralized nnU-Net Dice results.

### 3.5. External Generalization on CVC-ClinicDB

To examine cross-dataset generalization, the Kvasir-SEG-trained Baseline and Stage5-Swin models were directly evaluated on the external CVC-ClinicDB dataset. As shown in Table 9, Stage5-Swin achieved a Dice of 0.7895 and an IoU of 0.7033, which were slightly lower than the Baseline’s values of 0.7938 and 0.7075. Precision also decreased slightly from 0.8133 to 0.8057. However, Stage5-Swin obtained a slightly higher Recall value of 0.8549 compared with 0.8481 for the Baseline, and its HD95 was marginally lower, decreasing from 44.8380 to 44.7037.

These results indicate that Stage5-Swin maintains comparable external performance on CVC-ClinicDB and does not exhibit evident failure under external-domain transfer. Nevertheless, unlike the Kvasir-SEG clean and degradation experiments, the external CVC-ClinicDB evaluation does not support a claim that Stage5-Swin is comprehensively superior to the Baseline. Instead, Stage5-Swin shows slightly lower Dice, IoU, and Precision but slightly higher Recall and lower HD95, suggesting a different trade-off between region overlap, lesion sensitivity, and boundary stability under cross-dataset transfer. A paired per-image comparison further showed no statistically significant difference between the Baseline and Stage5-Swin across the five evaluation metrics on CVC-ClinicDB. Therefore, the CVC-ClinicDB experiment should be interpreted as preliminary evidence of cross-dataset stability rather than evidence of universal external-domain superiority.

### 3.6. Resource-Aware Efficiency, Model Size, and Communication Cost

Table 10 presents the computational efficiency, memory footprint, and estimated federated communication costs of the centralized models for resource-constrained edge-sensing scenarios. To ensure reproducible efficiency evaluation, all models were measured under the same inference protocol on an NVIDIA GeForce RTX 4090 GPU. The efficiency measurements were performed using CUDA 11.8 and PyTorch 2.1.2+cu118. Specifically, inference was performed with a 640 × 640 input resolution using FP32 Precision. FPS was calculated after 30 warm-up iterations and averaged over 100 repeated forward passes, excluding data loading and postprocessing time. Compared with the Baseline, Stage5-Swin increases the parameter count from 46.29 M to 49.45 M and FLOPs from 93.75 G to 95.02 G, while FPS decreases only from 148.23 to 144.51, and inference GPU memory remains nearly unchanged. Compared with Stage4-Swin, Stage5-Swin achieves better clean and degraded-test performance with lower FLOPs and higher FPS, suggesting a better accuracy–efficiency trade-off from a resource-aware perspective.

The communication overhead was theoretically estimated based on the model parameter count under a standard FedAvg communication process. Specifically, each communication round was assumed to include one server-to-client model transmission and one client-to-server model update transmission using float32 parameters. The bidirectional communication volume per client per round is estimated as follows:

(6) $$C_{round}=\frac{2\times P\times 4}{1024^{2}}\;\mathrm{MB},$$

where 2 represents two transfers, server-to-client and client-to-server, and 4 represents the number of bytes for a float32 parameter. The total communication volume for four clients over 20 rounds is further multiplied by the number of clients and the number of rounds. Since Stage4-Swin and Stage5-Swin have the same number of parameters, the two-way communication volume per client per round is 377.25 MB, and the total communication volume for four clients over 20 rounds is approximately 29.47 GB.

## 4. Discussion

The core finding is that Swin Transformer enhancement in nnU-Net v2 exhibits clear stage dependency. Stage7-Swin is located near the bottleneck, where the 5 × 5 feature map provides very few tokens for spatial self-attention. Stage4-Swin retains a larger 40 × 40 feature map, but its early insertion may interfere with mid-level convolutional feature extraction. This behavior may contribute to less stable boundaries on the independent test set. Stage5-Swin operates on a 20 × 20 middle-to-deep feature map and provides a better balance among semantic abstraction, token count, and computational cost. Although a complete exhaustive sweep of all encoder stages was not performed, the selected stages represent three distinct feature resolution regimes and were designed to capture the expected trade-off between spatial detail preservation and computational burden. This conclusion is consistent with recent calls for rigorous validation of medical segmentation models: new structural modules should be systematically evaluated against strong baselines, independent test sets, and computational cost constraints, rather than relying solely on a single validation set metric [3]. 

The robustness experiments showed that Stage5-Swin maintained a stable Dice-based relative advantage over the Baseline across the evaluated corruption–severity settings; however, the inferential results were metric- and corruption-dependent. Under moderate blur, Stage5-Swin significantly improved Dice, IoU, Precision, and HD95 relative to the Baseline. This pattern suggests that the middle-to-deep Swin module may use broader contextual information to compensate for weakened local appearance cues. Under moderate Gaussian noise, Stage5-Swin also achieved a significantly lower mean HD95, although the other four metrics did not reach the raw significance threshold. In contrast, moderate overexposure produced a significant Precision–Recall trade-off: Stage5-Swin increased Precision but reduced Recall. This result suggests a more conservative segmentation behavior under illumination saturation and a potentially greater risk of omitting lesion pixels. Other observed mean differences, including the higher JPEG HD95 for Stage5-Swin, were not statistically significant. Overall, these findings indicate that robustness improvements are corruption-, metric-, and case-dependent rather than universal. Because the 35 raw *p*-values were not corrected for multiple comparisons, these inferential findings should be regarded as exploratory and require confirmation in additional datasets or repeated experiments. Future work should particularly investigate overexposure-aware augmentation, brightness normalization, and methods that improve lesion coverage without sacrificing Precision.

For practical deployment, future work could combine brightness normalization, overexposure-enhanced training, or uncertainty-based warning mechanisms to further reduce the risk of missed detections. The external CVC-ClinicDB evaluation further clarifies the generalization behavior of Stage5-Swin. In contrast to the Kvasir-SEG clean and degradation experiments, where Stage5-Swin showed the best overall accuracy–robustness trade-off, the CVC-ClinicDB results demonstrate comparable rather than superior performance relative to the Baseline. Stage5-Swin obtained slightly lower Dice, IoU, and Precision but slightly higher Recall and lower HD95. This pattern suggests that the mid-to-deep Swin insertion remains usable under external-domain transfer and may help preserve lesion sensitivity and boundary stability to some extent. However, the marginal decrease in Dice and IoU also indicates that dataset-level appearance shifts still affect the model. Therefore, the external experiment should be interpreted as evidence of preliminary cross-dataset stability, not as proof of universal external-domain superiority.

The FedAvg experiment should be interpreted as a supplementary compatibility analysis rather than an additional performance claim. Both Stage4-Swin and Stage5-Swin successfully completed local training, weight aggregation, validation monitoring, and global model saving in the same four-client IID FedAvg workflow, indicating that the Swin-embedded nnU-Net v2 variants are trainable under this collaborative protocol [23,24,25,26,27]. The validation performance trajectories show that Stage5-Swin achieves a higher best validation pseudo-Dice than Stage4-Swin and approaches that of the FedAvg Baseline model, which is consistent with the centralized finding that Stage5 is a more suitable insertion position than Stage4. However, these federated results should not be interpreted as proof of federated superiority because the experiment is limited to an IID split and 20 communication rounds. Performance fluctuations remained near the end of the 20-round runs; thus, full convergence cannot be established. Therefore, the observed difference between centralized and federated performance may result from multiple factors, including limited communication rounds, distributed optimization characteristics, and differences between centralized and federated training protocols. It may instead reflect the optimization constraints introduced by distributed aggregation. Therefore, the federated results support workflow feasibility and training-trend visualization, whereas the main performance conclusions of this paper remain grounded in centralized independent testing and degradation robustness experiments. Further validation under non-IID data, cross-institutional feature shift, secure aggregation, differential privacy, and communication compression remains an important direction for future work [28,29].

In the context of intelligent communication, computing, and sensing, the results provide three complementary observations. The FedAvg experiment showed that the proposed Swin-embedded architecture could participate in a federated collaborative training workflow without exchanging raw endoscopic images. The degradation experiments evaluate sensing robustness under dynamic visual conditions caused by acquisition or transmission artifacts. The efficiency and communication cost analysis further connects the model design to resource-constrained edge-AI scenarios, where segmentation accuracy must be balanced against FLOPs, FPS, GPU memory, model size, and communication overhead. These aspects demonstrate the relevance of the proposed evaluation framework to collaborative and resource-aware endoscopic sensing, although the present work does not address multimodal sensing fusion.

This study has several limitations. First, although an initial external evaluation was conducted on CVC-ClinicDB, the current cross-dataset validation is still limited to a single external dataset. More diverse external endoscopy datasets and multicenter clinical data are needed to further evaluate generalization stability under broader domain shifts [4,5]. Second, centralized training primarily used fold 0 and a single random seed. Future work should conduct repeated experiments with multiple folds or multiple seeds to obtain more robust confidence intervals. Third, the federated experiments currently cover only a four-client IID setting with 20 communication rounds for the Baseline, Stage4-Swin, and Stage5-Swin models. They do not yet include non-IID feature distributions, real cross-institutional data heterogeneity, secure aggregation, differential privacy, or communication compression strategies. Furthermore, the corruption experiments were based on synthetic perturbations rather than real acquisition artifacts. Future work should investigate robustness using prospectively collected degraded endoscopic sequences or real-world acquisition artifacts from multicenter clinical data.

Overall, the results indicate that the proposed framework should be interpreted as a stage-aware and resource-aware evaluation of Swin insertion within nnU-Net v2 rather than as a general claim that attention modules always improve medical segmentation. Therefore, this study is not intended as a direct benchmark competition with existing SOTA polyp segmentation models, such as PraNet, HarDNet-MSEG, UACANet, Polyp-PVT, or ColonFormer. These methods employ different customized architectures and optimization strategies, whereas our objective is to isolate the effect of Swin insertion stages within a fixed nnU-Net v2 framework. Such a controlled comparison enables clearer interpretation of how Transformer placement influences robustness, generalization, and computational trade-offs. The centralized and degradation experiments indicate that Stage5 provides the most balanced insertion point under the current Kvasir-SEG 2D setting because it combines sufficient semantic abstraction with a still-meaningful spatial token grid. The degradation analysis further shows that robustness depends on both the corruption type and the evaluation metric. Stage5-Swin showed the clearest multi-metric improvements under moderate blur, together with significant improvements in low-resolution Precision and noise HD95. In contrast, moderate overexposure revealed a significant Precision–Recall trade-off. The FedAvg experiment demonstrates that the Swin-embedded variants can be trained in a collaborative workflow without exchanging raw images, but it remains a preliminary IID feasibility analysis rather than evidence of federated superiority. The efficiency analysis showed that Stage5-Swin introduced a moderate additional cost relative to the Baseline and remained more efficient than Stage4-Swin. However, practical edge deployment requires further validation. Future studies should include latency measurements on real edge hardware, non-IID federated evaluation, privacy mechanisms, secure aggregation, and communication compression.

## 5. Conclusions

This study developed a stage-aware Swin-enhanced nnU-Net v2 framework for colon polyp segmentation in intelligent endoscopic sensing. The framework was evaluated from four perspectives: centralized segmentation performance, degradation robustness, federated collaborative training, and resource-related cost. In colonoscopy, reliable polyp segmentation is important because endoscopic images are affected by variable illumination, motion, compression, and lesion appearance changes during acquisition. Therefore, a segmentation framework that considers robustness and computational constraints is more relevant to practical endoscopic visual sensing than a model evaluated only under ideal image conditions. The results demonstrated that the insertion stage of the Swin Transformer substantially affects model behavior. Under the current Kvasir-SEG 2D configuration, Stage5-Swin achieved the most favorable overall trade-off among the evaluated insertion positions. Its middle-to-deep placement retained sufficient semantic abstraction while preserving a meaningful spatial token grid. This design is potentially beneficial for colonoscopic images, where polyps may exhibit ambiguous boundaries and heterogeneous appearance, because it introduces contextual modeling without completely replacing the original nnU-Net feature extraction pipeline.

Stage5-Swin maintained the highest Dice across all evaluated corruption–severity combinations. However, the inferential improvements were not uniform across all metrics, conditions, or individual cases. Moderate blur showed the clearest multi-metric statistical improvements, and moderate Gaussian noise showed a significant aggregate reduction in HD95. Nevertheless, representative noise-degraded cases demonstrated that slightly improved Dice could still coexist with worse per-image HD95 because of localized boundary outliers. In contrast, moderate overexposure produced higher Precision but lower Recall, indicating a separate condition-specific trade-off. External evaluation on CVC-ClinicDB showed performance comparable to the Baseline rather than consistent superiority. The supplementary FedAvg experiment demonstrated compatibility with multi-client collaborative training, but it was limited to a four-client IID setting. Resource analysis showed that Stage5-Swin introduced a moderate computational and communication overhead.

Despite these advantages, several challenges remain before practical clinical implementation. First, the current validation is based on publicly available datasets rather than prospective multicenter colonoscopy data; therefore, robustness under real clinical variations, including different endoscopic devices, operators, and patient populations, remains uncertain. Second, the corruption experiments provide controlled robustness assessment but cannot fully reproduce complex acquisition conditions in real colonoscopy. Third, although the proposed model introduces only moderate additional computational cost, real-time deployment requires further validation on clinical endoscopy systems or edge hardware. In addition, the federated experiment was limited to an IID setting and did not consider realistic cross-institutional heterogeneity, privacy-preserving mechanisms, or communication constraints.

These findings support stage-aware model design for degradation-aware endoscopic segmentation. Nevertheless, they do not constitute direct clinical or edge-device deployment validation. Future work should include additional external datasets, multicenter clinical data, repeated training with multiple folds or seeds, non-IID federated settings, communication compression, privacy mechanisms, and latency testing on real edge hardware.

## Figures and Tables

**Table 1:** Dataset, training configuration, and unified evaluation settings.

Item	Setting
Dataset	Kvasir-SEG, Dataset001_Kvasir
Training and validation	900 images, fold 0 split: 720 training/180 validation
Independent clean test	100 images, imagesTs/labelsTs/case_mapping.csv
nnU-Net configuration	2D, patch size 640 × 640, batch size 8
Optimizer schedule	nnU-Net default training strategy (1000 epochs), initial learning rate 0.01
Randomness control	seed = 42, identical plans and split files for the Baseline and Swin variants
Evaluation metrics	Dice, IoU, Precision, Recall, HD95
Environment	NVIDIA GPU, 48 GB VRAM, Python 3.10, PyTorch 2.1.2, CUDA 11.8, nnU-Net v2

Note: IoU = intersection over union; HD95 = 95th percentile Hausdorff distance; VRAM = video random-access memory. The folders imagesTs and labelsTs denote the independent test images and labels in the nnU-Net v2 data structure, respectively.

**Table 2:** Stage-aware Swin insertion configurations in the nnU-Net v2 PlainConvUNet encoder.

Variant	Insertion Position	Feature Map	Channel	Rationale
Baseline	None	-	-	Original nnU-Net v2 2D model
Stage4-Swin	After stage 4	40 × 40	512	More tokens, stronger spatial modeling, and higher computational cost
Stage5-Swin	After stage 5	20 × 20	512	Middle-to-deep semantic features and moderate token count
Stage7-Swin	After stage 7	5 × 5	512	Bottleneck-like insertion, very few tokens

Note: Stage indices follow the zero-based indexing used in the nnU-Net v2 PlainConvUNet encoder. Therefore, “stage 4”, “stage 5”, and “stage 7” correspond to the outputs after encoder.stages [4], encoder.stages [5], and encoder.stages [7], respectively. Map size denotes the feature map resolution under the 640 × 640 2D input configuration.

**Table 3:** Centralized ablation results on a 100-image independent clean test set.

Model	Dice	IoU	Precision	Recall	HD95
Baseline	0.8838	0.8222	0.8807	0.9226	43.1908
Stage4-Swin	0.8773	0.8145	0.8787	0.9266	50.9674
Stage5-Swin	0.8876	0.8245	0.8871	0.9276	41.7450
Stage7-Swin	0.8743	0.8063	0.8721	0.9187	58.2690

Note: Higher Dice, IoU, Precision, and Recall values indicate better segmentation performance, whereas lower HD95 values indicate smaller boundary error.

**Table 4:** Average robustness performance across six moderate-severity degraded test sets.

Model	Dice	IoU	Precision	Recall	HD95	Dice Drop	IoU Drop	HD95 Inc
Baseline	0.8771	0.8132	0.8733	0.9218	46.99	0.0067	0.009	3.8
Stage4-Swin	0.8737	0.8093	0.873	0.9249	53.17	0.0036	0.0052	2.2
Stage5-Swin	0.8843	0.8201	0.8848	0.9237	45.54	0.0033	0.0044	3.79

Note: Averages are computed over the six degraded test sets, excluding the clean test set. Dice Drop is calculated as clean Dice minus degraded Dice, IoU Drop as clean IoU minus degraded IoU, and HD95 Increase as degraded HD95 minus clean HD95. Higher Avg Dice, Avg IoU, Avg Precision, and Avg Recall values are better, whereas lower Avg HD95, Avg Dice Drop, Avg IoU Drop, and Avg HD95 Increase values indicate better robustness. HD95 Inc = Average HD95 Increase. The moderate levels correspond to Gaussian noise σ=5, box blur radius 2, low-illumination factor 0.6, overexposure factor 1.4, JPEG quality 30, and low-resolution scale 0.5.

**Table 5:** Dice scores under different corruption types and severity levels.

Corruption	Severity	Baseline	Stage4-Swin	Stage5-Swin
Noise	Mild	0.883	0.878	**0.888**
Noise	Moderate	0.881	0.872	**0.885**
Noise	Severe	0.867	0.861	**0.874**
Blur	Mild	0.884	0.877	**0.888**
Blur	Moderate	0.881	0.877	**0.889**
Blur	Severe	0.867	0.858	**0.872**
JPEG	Mild	0.877	0.871	**0.885**
JPEG	Moderate	0.882	0.878	**0.885**
JPEG	Severe	0.850	0.821	**0.854**
Low-light	Mild	0.884	0.877	**0.888**
Low-light	Moderate	0.884	0.877	**0.888**
Low-light	Severe	0.884	0.878	**0.887**
Overexposure	Mild	0.869	0.873	**0.881**
Overexposure	Moderate	0.851	0.861	**0.870**
Overexposure	Severe	0.823	0.839	**0.840**
Low-resolution	Mild	0.884	0.877	**0.888**
Low-resolution	Moderate	0.883	0.878	**0.889**
Low-resolution	Severe	0.875	0.873	**0.887**

Note: All results were obtained on the same 100-image independent Kvasir-SEG test set. Higher Dice values indicate better segmentation performance. For each corruption type, mild, moderate, and severe denote progressively stronger corruption intensities defined in Section 2.4. The best value in each row is shown in bold.

**Table 6:** Representative significant differences between the Baseline and Stage5-Swin under moderate-degradation conditions based on two-sided paired Wilcoxon signed-rank tests.

Scenario	Metric	Baseline Mean	Stage5 Mean	Difference	*p*-Value
blur	Dice	0.8815	0.8886	0.0071	0.0177
blur	IoU	0.8185	0.8266	0.0081	0.0169
blur	HD95	44.7238	41.6485	−3.0753	0.0106
blur	Precision	0.8757	0.8856	0.0099	0.0381
low_resolution	Precision	0.8781	0.8869	0.0088	0.045
noise	HD95	44.2674	41.042	−3.2254	0.0268
overexposure	Precision	0.8478	0.8792	0.0314	0.0316
overexposure	Recall	0.9189	0.9028	−0.0161	0.0404

Note: Two-sided paired Wilcoxon tests (case_id-matched); only *p* < 0.05 shown. Positive diff favors Stage5-Swin; negative HD95 diff = better. Full results are provided in Table S1.

**Table 7:** FedAvg experimental setup and model selection.

Item	Baseline FedAvg	Stage4-Swin FedAvg	Stage5-Swin FedAvg
Clients	4	4	4
Distribution	IID	IID	IID
Rounds	20	20	20
Local epochs	1	1	1
Iterations per epoch	50	50	50
Validation iterations	10	10	10
Best round	18	16	19
Final round	19	19	19

Note: Round numbering starts from 0; therefore, round 19 denotes the 20th and final communication round. The best round was selected according to the highest mean validation pseudo-Dice recorded in federated_training_metrics.csv. For Stage5-Swin, the best validation checkpoint coincides with the final round.

**Table 8:** Evaluation results of the FedAvg global model on a test set of 100 clean images.

Model	Round Type	Dice	IoU	Precision	Recall	HD95
FedAvg Baseline	Best R18	0.6895	0.5761	0.6864	0.8087	128.69
FedAvg Stage4-Swin	Best R16	0.6511	0.5350	0.6542	0.7729	133.85
FedAvg Baseline	Final R19	0.7125	0.5998	0.7056	0.8302	118.76
FedAvg Stage4-Swin	Final R19	0.6999	0.5933	0.7017	0.8019	110.41
FedAvg Stage5-Swin	Best/Final R19	0.6792	0.5677	0.6909	0.7728	127.47

Note: Best R18 and Best R16 denote the global model checkpoints selected according to the highest mean validation pseudo-Dice for the corresponding method, whereas Final R19 denotes the final saved communication round. Higher Dice, IoU, Precision, and Recall values indicate better performance, while lower HD95 values indicate smaller boundary error.

**Table 9:** External evaluation results on CVC-ClinicDB.

Model	Dice	IoU	Precision	Recall	HD95
Baseline	0.7938	0.7075	0.8133	0.8481	44.838
Stage5-Swin	0.7895	0.7033	0.8057	0.8549	44.7037

Note: Models were trained on Kvasir-SEG and directly evaluated on all 612 CVC-ClinicDB images without additional fine-tuning.

**Table 10:** Model efficiency, model size, and FedAvg communication overhead.

Model	Params (M)	FLOPs (G)	FPS	GPU Mem (MiB)	Checkpoint Size (MiB)	Cost/ Client (MB)	Total Comm (GB)
Baseline	46.29	93.75	148.23	3207.24	353	353.19	27.59
Stage4-Swin	49.45	98.83	138.89	3227.84	377	377.25	29.47
Stage5-Swin	49.45	95.02	144.51	3227.40	377	377.25	29.47

Note: Params are reported in millions of trainable parameters, and FLOPs are reported in billions under the same 2D input configuration. FPS was measured using the inference protocol described in Section 3.6. GPU memory denotes peak allocated memory during inference with a batch size of 8. Checkpoint size represents the measured size of checkpoint_best.pth.

## Data Availability

The Kvasir-SEG and CVC-ClinicDB datasets analyzed in this study are publicly available and are described in [4] and [5], respectively. The code, model configurations, and evaluation scripts supporting the findings of this study are available from the corresponding author upon reasonable request due to ongoing code organization and institutional project management requirements.

## Supplementary Materials

The following supporting information can be downloaded at: https://www.mdpi.com/article/10.3390/s26165206/s1, Table S1: Complete two-sided paired Wilcoxon signed-rank test results between the Baseline and Stage5-Swin across clean and degraded scenarios.

## Abbreviations

The following abbreviations are used in this manuscript:

CNN	Convolutional Neural Network
FedAvg	Federated Averaging
FLOPs	Floating-Point Operations
FPS	Frames Per Second
GPU	Graphics Processing Unit
HD95	95th Percentile Hausdorff Distance
IID	Independent and Identically Distributed
IoU	Intersection Over Union
