# Supplementary material for: Stage-Aware Swin-Enhanced nnU-Net v2 for Robust Polyp Segmentation in Collaborative Endoscopic Visual Sensing
Source: Sensors (Basel). 2026 Aug 17;26(16):5206. doi: 10.3390/s26165206 (PMC13517513; doi:10.3390/s26165206)
Supplement: Supplementary file 1 [file sensors-26-05206-s001.zip › sensors-4425628-supplementary.pdf]

Supplementary Table S1. Complete two-sided paired Wilcoxon signed-rank test results between the Baseline and Stage5-Swin across clean and degraded scenarios.

Scenario	Metric	Baseline Mean	Stage5-Swin Mean	Difference	p-value	Significance	Trend
clean	Dice	0.8838	0.8876	0.0038	0.401497	ns	Improved
clean	IoU	0.8222	0.8245	0.0023	0.362213	ns	Improved
clean	HD95	43.1908	41.745	-1.4458	0.067466	ns	Improved
clean	Precision	0.8807	0.8871	0.0064	0.235535	ns	Improved
clean	Recall	0.9226	0.9276	0.005	0.539023	ns	Improved
blur	Dice	0.8815	0.8886	0.0071	0.017671	*	Improved
blur	IoU	0.8185	0.8266	0.0081	0.016865	*	Improved
blur	HD95	44.7238	41.6485	-3.0753	0.010637	*	Improved
blur	Precision	0.8757	0.8856	0.0099	0.038143	*	Improved
blur	Recall	0.9236	0.9298	0.0062	0.950651	ns	Improved
jpeg	Dice	0.8819	0.8845	0.0026	0.799159	ns	Improved
jpeg	IoU	0.819	0.8202	0.0012	0.909662	ns	Improved
jpeg	HD95	44.9511	46.0739	1.1228	0.632002	ns	Worse
jpeg	Precision	0.8778	0.8836	0.0058	0.136541	ns	Improved
jpeg	Recall	0.9225	0.9275	0.005	0.062382	ns	Improved
low_light	Dice	0.8838	0.8876	0.0038	0.40731	ns	Improved
low_light	IoU	0.8221	0.8245	0.0024	0.373183	ns	Improved
low_light	HD95	43.226	41.8768	-1.3492	0.077666	ns	Improved
low_light	Precision	0.8807	0.887	0.0063	0.238261	ns	Improved
low_light	Recall	0.9225	0.9277	0.0052	0.518017	ns	Improved
low_resolution	Dice	0.883	0.889	0.006	0.081293	ns	Improved
low_resolution	IoU	0.8208	0.8268	0.006	0.074344	ns	Improved
low_resolution	HD95	43.32	41.6379	-1.6821	0.066834	ns	Improved
low_resolution	Precision	0.8781	0.8869	0.0088	0.045012	*	Improved
low_resolution	Recall	0.9237	0.9283	0.0046	0.687475	ns	Improved

Scenario	Metric	Baseline Mean	Stage5-Swin Mean	Difference	p-value	Significance	Trend
noise	Dice	0.881	0.8855	0.0045	0.250803	ns	Improved
noise	IoU	0.818	0.8221	0.0041	0.219641	ns	Improved
noise	HD95	44.2674	41.042	-3.2254	0.026779	*	Improved
noise	Precision	0.8795	0.8868	0.0073	0.262333	ns	Improved
noise	Recall	0.9194	0.9261	0.0067	0.874328	ns	Improved
overexposure	Dice	0.8512	0.8704	0.0192	0.540523	ns	Improved
overexposure	IoU	0.7804	0.8007	0.0203	0.518017	ns	Improved
overexposure	HD95	61.4399	60.9324	-0.5075	0.703599	ns	Improved
overexposure	Precision	0.8478	0.8792	0.0314	0.031638	*	Improved
overexposure	Recall	0.9189	0.9028	-0.0161	0.040438	*	Worse

Note: Two-sided paired Wilcoxon signed-rank tests were performed using case_id matched samples.
